# Towards national health insurance: Alignment of strategic human resources in South Africa

**DOI:** 10.4102/phcfm.v11i1.1928

**Published:** 2019-06-24

**Authors:** Nathaniel Mofolo, Christo Heunis, Gladys N. Kigozi

**Affiliations:** 1 School of Clinical Medicine, University of the Free State, Bloemfontein, South Africa; 2 Centre for Health Systems Research and Development, University of the Free State, Bloemfontein, South Africa

**Keywords:** health policy, national health programmes, primary health care, health care workers, health care disparities, state, government

## Abstract

**Background:**

South Africa is implementing national health insurance (NHI) and primary health care (PHC) re-engineering, and has concomitantly introduced the Human Resources for Health (HRH) Strategy. These policies are underpinned by the National Development Plan (NDP), which aims to address widespread inequality and inequity.

**Aim:**

The aim of this study was to analyse the alignment of national HRH-related policies to implement NHI and PHC re-engineering and determine knowledge gaps and research needs.

**Method:**

A narrative review of the NDP, PHC re-engineering, HRH and NHI strategies was carried out, supplemented by key HRH reports, data and articles.

**Results:**

Current policies stress NHI and PHC re-engineering without effectively addressing shortages and maldistribution of HRH across the provincial and public–private divides. In line with PHC re-engineering, the HRH Strategy emphasised strengthening of community health workers (CHWs), professional nurses (PNs), mid-level workers (MLWs), medical practitioners (MPs) and clinical specialists (CSs). Four of these, CHWs, MLWs, MPs and CSs, are varyingly still in absolute shortfall, as well as being inequitably distributed across the provincial and public–private divides. The seeming adequacy in the absolute number of PNs may disguise provincial and public–private sector disparities. Although expedited HRH development and equitable deployment are crucial, it is also vital to resolve extant education and accreditation challenges delaying HRH policy implementation.

**Conclusion:**

The current lack of alignment of HRH policies does not portend well for the successful implementation of NHI and PHC re-engineering. Knowledge gaps include the need for further clarification of ideal multi-disciplinary team compositions and responsibilities.

## Introduction

Difficulties in the financing, training, deployment, retention and performance of human resources for health (HRH) are global challenges.^1,2^ In 2013, the worldwide deficit of health care workers (HCWs) was about 17.4 million, including 2.6 million medical doctors or practitioners (MPs) and 9.0 million nurses and midwives.^1^ South Africa’s unique HRH challenges stem from a two-tiered public–private order that produces highly inequitable health care access and outcomes.^3,4^ Half of the national health resources are spent on 16% of the population covered by medical schemes, while the other half are spent on the 84% public sector-reliant population.^5^

Unremitting health care inequality and inequity contradicts the aim of the *National Health Act* (61 of 2003)^6^ to transform the national health system. The Act requires the National Health Council to monitor HRH utilisation across the public and private sectors. It also provides for a district health system (DHS) with municipalities responsible for their own primary health care (PHC) budgets. Primary health care clinics are the entry point for patients in need of preventative care and diagnosis and treatment of minor ailments. Professional nurses (PNs) with additional PHC training usually render these services, with MPs visiting on a rotating basis.

Launched in 2012, the National Development Plan (NDP)^7^ is the country’s blueprint for sustainable growth until 2030. One of the ‘critical actions’ of the Plan is to phase in national health insurance (NHI). The 2017 National Department of Health’s (NDoH) White Paper^8^ envisaged that NHI will bridge the gap between the public and private sectors. National health insurance is being phased in over a 14-year period, with the first phase (2012–2017), which focused on public sector strengthening, already in the past.

The NDoH’s HRH Strategy for Health (2011)^9^ mainly emphasised the strengthening of the following types of HCWs in line with PHC re-engineering: community health workers (CHWs), PNs, MPs and clinical specialists (CSs) (community-based), as well as mid-level workers (MLWs) (district hospital-based). Primary health care re-engineering aims to improve community health through three streams: ward-based outreach teams (WBOTs), school health services and district clinical specialist teams (DCSTs).^10^ The DCSTs were necessitated by the high maternal mortality in the country, that is, almost six in every 10 maternal deaths were potentially preventable.^11^ The WBOTs are managed by PNs who are additionally responsible for post-natal and CHW-referred home visits to patients.^12^ The HRH Strategy also described other workforce implications of PHC re-engineering such as the need to re-establish the MP’s function as a key clinical role player in the PHC team.

### Aim

This study analyses the alignment of South Africa’s latest HRH-related policies to give effect to the critical mass and combinations of HCWs required by NHI and PHC re-engineering. Specific review questions were, firstly, to determine the level of congruence between the NDP (2012), PHC re-engineering (2011), HRH (2011) and NHI (2017) policies; secondly, to describe the current and required numbers of CHWs, PNs, MLWs, MPs and CSs and thirdly, to identify knowledge gaps and areas for research.

## Methods

Information to answer the pre-determined research questions was sought by means of a narrative review of secondary documents conducted by all authors independently with subsequent discussions to reach consensus on the relevance and implications of each document. The main documents reviewed include the government’s (1) NDP (2012)^7^ as well as the NDoH’s (2) PHC re-engineering (2011),^10^ (3) HRH (2011)^9^ and (4) NHI (2017)^8^ policies. The supplementary policies, strategies, reviews, reports and databases reviewed include chapters of the Health Systems Trust’s (HST) South African Health Reviews of (1) 2014–2015,^13,14,15^ (2) 2016,^16^ and (3) 2017^17^; the World Health Organization’s (WHO) (4) Evidence on CHW Programmes (2007),^18^ (5) Recommendations to Optimize HCW Roles via Task Shifting (2012),^19^ (6) Health Statistics to Monitor the Sustainable Development Goals (SDGs) (2017)^2^ and (7) Global Health Observatory data repository (2018)^20^; the Global Health Workforce Alliance’s (GHWA) report (8) CHWs for the Millennium Development Goal (MDG) Delivery (2010)^21^; the Medical Research Council’s (MRC) (9) PHC Re-engineering Staffing Norms (2012)^12^; the NDoH’s (10) Policy on Recruitment and Employment of Foreign Health Professionals (c2006),^22^ (11) Strategic Plan for Nursing (2012),^23^ and (12) Policy Framework and Strategy for WBOTs (2018/19–2023/24)^24^; the Competition Commission’s (13) Health Market Inquiry (HMI) (2018)^25^; Econex’s report, (14) Doctor Shortages (2015)^26^; the Foundation for Professional Development’s (FPD) report, (15) Role of Doctors in a Public Sector PHC Team (2017)^27^; the Clinical Associate National Task Team’s report, (16) CA Training and Profession (2017)^28^; and the Albertina Sisulu Executive Leadership Programme in Health’s (ASELPH) (17) Rapid Appraisal of WBOTs (2015).^29^

## Findings

South Africa’s transition to democracy since 1994 included the introduction of free health care policies, the DHS and prioritisation of PHC, which all contributed to ensure more equal distribution of HCWs as well as greater access to services.^30^ However, district-based PHC implementation was confronted with the emergence of a human immunodeficiency virus (HIV) and/or acquired immune deficiency syndrome (AIDS)-tuberculosis (TB) co-epidemic, as well as a rising burden of chronic non-communicable diseases (NCDs).^31^ This drastically increased the need for public health services. However, constant emigration of health professionals to wealthier countries and to the well-resourced private sector undermined the strength of the public sector.

In 2016, Rispel^16^ noted that South Africa had higher ratios of health professionals than the WHO minimum norms and also had ‘well-established training institutions (among the best in the world), highly skilled [HCWs], effective professional regulation, and sufficient fiscal space for relatively high remuneration levels compared with many other countries’. Nonetheless, different types of HRH crises were occurring in the country, including a staff-shortage crisis with more than 100 000 public sector vacancies in 2010.

The MRC’s PHC Re-engineering Staffing Norms (2012)^12^ provided the following counsellor (CHW) and health professional and staffing norms per 10 000 client visits expressed as the required number of full-time equivalent posts: CHWs – 0.63, PNs – 1.52 and MPs – 0.08. When applying these norms to the actual PHC utilisation rate, there were shortfalls of 1652 CHWs, over 10 000 PNs and 361 MPs in the public sector. Thereupon, assuming a ratio of one CHW to 600 people, a civil society publication^32^ estimated that over 60 000 CHWs were needed. The NDoH’s HRH Strategy (2011)^9^ indicated the numbers of public sector vacancies for PNs as 44 780 and MPs as 10 860. These differences show that the extent of HCW shortages is debatable, depending on the information that is used. Still, as indicated by the WHO’s Health Statistics for the SDGs (2017),^2^ at 5.88 per 1000 population, South Africa’s skilled health professional density over the years 2005–2015 was higher than that globally at 4.56 per 1000 population. Skilled birth assistance in South Africa over the years 2005–2016 at 94% was higher than the 81% of India, but lower than the 100% of China and the Russian Federation, respectively, and the 99% of Brazil. Female life expectancy in South Africa in 2015 was 66.2 years, that is, substantially lower than 73.7 years globally, 78.7 years in Brazil, 76.3 years in the Russian Federation and also somewhat lower than the 69.9 years in India. Interestingly, at 2.75 per 1000 population, India had less than half of South Africa’s skilled health professional density. Current HRH challenges in South Africa pertain especially, but not exclusively, to CHWs, PNs, MLWs, MPs and CSs as elaborated below.

### Community health workers

The WHO’s Recommendations to Optimize HCW Roles via Task Shifting (2012)^19^ viewed rational distribution of tasks among cadres of HCWs as a promising strategy to improve access and cost-effectiveness within health systems. The GHWA report, CHWs for MDG Delivery (2010)^21^ showed that CHWs provided a wide variety of services to communities, contributed to the lowering of maternal and child mortality rates, and assisted in decreasing the burden and costs of communicable diseases such as TB and malaria. A renewed focus on a more diverse skills mix and a greater role for CHWs may result in an increase in their numbers relative to the number of professional HCWs in the future.^1^

The WHO’s Global Health Observatory (2018)^20^ combines community and traditional health workers in a single category making country comparisons of the number of CHWs difficult. For some countries, including Brazil and the Russian Federation, no relevant statistics are provided. The ratio of 0.192 for traditional and CHWs per 1000 population indicated for South Africa dates back to 2004. To the extent comparable, this was substantially lower than the 0.581 per 1000 population in India and the 0.831 per 1000 population in China in 2011.

The NDP (2012)^7^ envisaged that PHC services will be strengthened by broadening district-based programmes such as the CHW programme. The HRH Strategy (2011)^9^ acknowledged that while the role of the CHW was central to the PHC initiative, historically this role was inadequately defined in South African HRH policy. Efforts are now under way to recognise CHWs as formal HCWs. To better integrate them into the general health system and existing community structures, task shifting to CHWs should be considered in relation to the roles of other HCWs.^33^ The HRH Strategy directed that the scopes of practice of all health professionals need to be revised to shift tasks to the category of HCW (including CHWs) that can perform the work most efficiently. Evidence suggests that CHWs are best suited to strengthen the demand side (e.g. to increase the number of women seeking antenatal care and skilled birth assistance), promote health and prevent illness, and encourage appropriate referral for minor ailments and injuries.^9^

ASELPH’s Rapid Appraisal of WBOTs (2015)^29^ revealed many challenges pertaining to CHW development, including that the organisation and timing of available training (particularly the need to complete phase 1 before starting work) was inadequate, slow progression through the training phases, inadequate instruction materials, unconducive learning spaces and budgetary constraints. The WBOT Policy Framework and Strategy (2018)^24^ also indicated that conditions in some geographical areas made it difficult for the WBOTs to cover the expected number of households. Also, a lack of uniforms and nametags compromised CHWs’ identification and acceptance by communities: ‘Community-based health workers are neither workers nor volunteers’.^24^ The Framework and Strategy reaffirmed plans to fully integrate CHWs into the formal health service delivery platform.

Despite recognition that CHWs are a critical resource for comprehensive PHC, there are few data available on their supply and distribution in South Africa.^13^ The HRH Strategy (2011)^9^ indicated a shortage of 14 279 CHWs and 9874 home-based care workers in 2015. The MRC’s (2012) PHC Re-engineering Staffing Norms calculated a need for 44 792 CHWs.^12^ This was based on the following assumptions: a population of 6000 per WBOT, eight home visits per day per CHW, five CHWs per team, 300 households per CHW and a total of 6999 teams. In 2014, the media^34^ indicated the numbers of ‘ward-based’ CHWs as follows: Eastern Cape – 4284 (42.3%), North West – 1872 (18.5%), Gauteng – 1062 (10.5%), KwaZulu-Natal – 846 (8.4%), Free State – 678 (6.7%), Limpopo – 546 (5.4%), Northern Cape – 486 (4.8%) and Mpumalanga – 348 (3.4%). These data, which excluded the Western Cape Province because of ‘a separate provincial system’, suggested that CHWs were inequitably distributed with just more than two-fifths of CHWs located in the Eastern Cape. In the same year, this province accommodated only 15.3% of the total public sector-dependent population.^13^ Thereupon, Gauteng with 23.3% and KwaZulu-Natal with 23.5% of the public sector-dependent population, both only had about one-tenth of the country’s CHWs.

### Professional nurses

Although the NDP (2012)^7^ envisaged the training of ‘more nurses’, the HRH Strategy (2011)^9^ indicated that a shortage of 22 121 nurses would occur in 2015. Ten years before, in 2005, the number of nurses in South Africa at 3.4 per 1000 population far exceeded the WHO’s minimum norm of 2.0 per 1000 population.^35^ The WHO’s Global Health Observatory (2018)^20^ indicates that at 5.2 nurses and midwives per 1000 population in 2016, these health professionals were less well supplied in South Africa than in two of the BRICS countries, the Russian Federation, 8.7 per 1000 population (2015) and Brazil, 7.4 per 1000 population (2013). As measured by healthy life expectancy in 2016, the need for nurses and midwives was, however, highest in South Africa (55.7 years), followed by the Russian Federation (63.5 years) and Brazil (66.0 years).^2^ In 2015, as with around half of all WHO member countries, two of the BRICS countries – China, 2.3 per 1000 population (2015) and India, 2.1 per 1000 population (2016) – had less than 3.0 nurses and midwives per 1000 population. The seeming adequacy of South Africa’s nursing workforce, however, disguises disparity in public–private distribution. The Competition Commission’s HMI (2018)^25^ indicated that of a total of 149 381 nurses in the country in 2015, 109 477 (73.3%) were employed in the public sector and 39 904 (26.7%) in the private sector.

Before the closure of the nursing colleges in South Africa in the 1990s,^36^ basic nursing training entailed a 4-year general diploma at a nursing college in a training hospital. Taking place in the ward from day 1, the training was practical and produced confident, caring and skilled nurses who then completed a further year in midwifery, psychiatry or community nursing. However, the course was changed to 4 years at a university or university-linked college. The repercussion is expensive theoretical training, with nurses often having little confidence, poor attitudes and many abandoning the profession.

The Strategic Plan for Nurse Education Training and Practice (2012)^22^ required specific competencies for nurses and legislative changes for a revised scope of practice. However, the nursing profession remains plagued with challenges with respect to education, staffing and regulation.^37^ As early as 2015, Rispel and Bruce^14^ warned: ‘A major crisis is looming unless issues of curriculum quality and relevance, nurse educator quality, educational resources, and governance of nursing education are addressed’. The NDoH has been planning to provide nursing staffing norms for the last 5 years, but has not yet done so. All higher education institution (HEI) nursing programmes need to be aligned to place nursing on a par with other health professions within a unitary educational system.^38^ Public nursing colleges producing about 80% of graduates need more time to fulfil the criteria to be accredited as HEIs. The higher education sector and South African Nursing Council (SANC) are slow in accrediting nursing programmes. Moreover, poor basic public schooling in the country fails to produce students able to cope with the high demands of the nursing curriculum.^39^

### Mid-level workers

In 2008, Lehmann^40^ indicated a need to increase knowledge about MLWs and their role in and impact on health outcomes internationally. The clinical associate (CA) was introduced in South Africa with the first group of students graduating in 2010. The introduction of this cadre was a government-initiated strategy to address the shortages of health professionals, particularly in rural areas.^41^ In 2017, the CA National Task Team^27^ reported that CA students were being trained and worked in district hospitals as PHC generalists providing care, treatment and medical services to people living with HIV. Unlike clinically trained (PHC) nurses who practice independently, CAs work under the supervision of an MP, but this can change over time as the CA gains experience.^42^ Ideally, CAs will perform many of the routine tasks consuming the MPs’ time, allowing them to focus on more complex work.^43^

The CA National Task Team’s Report (2017)^28^ identified challenges relating to funding of new posts to accommodate CA graduates, creating career paths to prevent brain drain once graduates have repaid their bursaries, and lack of clarity about their roles relative to other clinical staff. Only 794 CAs completed training at three universities in South Africa from 2010 to 2016, of which 720 commenced practice locally. The HRH Strategy^9^ estimated that about 1350 CAs are required for district hospitals (five per district hospital). There was, thus, a substantial shortfall in production, and the need remains to substantially increase CA training at no risk of overproduction.

Mid-level worker programmes sometimes fail as a result of a lack of synergy between the processes of regulation, production and employment.^42^ It seems that the CA programme in South Africa may suffer the same fate. Formalising and expanding the training of MLWs at HEIs has never even reached the planning and development phase. The HEIs need to be informed about the requirements for MLWs based on service plans, how training will be financed and the plans for employment and career pathing.

Despite the appreciation for CAs,^28,41,42,43^ this new cadre has also generated serious concerns. According to a 2017 Parliamentary Monitoring Group report,^44^ CAs were ‘ignored in rural areas’ and ‘poached by the United Kingdom’. In 2016, the scope of practice for a CA was promulgated by the Health Professions Council of South Africa, but has yet to be signed off by the Health Minister. This implies that it remains difficult to define MLWs’ roles within multi-disciplinary teams.

### Medical practitioners

Although the NDP (2012)^7^ envisaged ‘[i]ncreasing the numbers of medical professionals’, the HRH Strategy (2011)^9^ indicated that a shortage of 3930 MPs would occur in 2015. The WHO’s Global Health Observatory (2018)^20^ shows that over 45% of member states, including South Africa, have less than 10 MPs per 1000 population. Compared to the other BRICS countries, the supply of MPs at 8.2 per 1000 population in South Africa in 2016 was somewhat higher than India at 7.6 per 1000 population (2016), but substantially lower than the 39.8 per 1000 population in the Russian Federation (2015), 18.5 per 1000 population in Brazil (2013) and 18.1 per 1000 population in China (2015). Thereupon, as measured by healthy life expectancy at birth for both sexes in 2015, the need for MPs was highest in South Africa (62.9 years) and lowest in the Russian Federation (70.5 years) and Brazil (75.0 years).

The FPD’s report, Doctors’ Roles in Multi-disciplinary PHC Teams (2017),^27^ indicated elaborate roles for MPs, including, *inter alia*, to provide holistic care to patients, contribute to health promotion, provide clinical support and governance, mentor and support other team members, ensure continuity of care through effective referral, advocate for the community and improve health outcomes through an understanding of the social determinants of health. However, the Competition Commission’s HMI (2018)^25^ indicated that of a total of 20 067 general practitioners in the country in 2015, 11 299 (56.3%) were working in the public sector and 8768 (43.7%) in the private sector. Of the 11 827 medical specialists, 4233 (35.8%) were working in the public sector and 7595 (64.3%) in the private sector. The comparatively few available MPs in South Africa were further maldistributed across the provinces, with an overconcentration of medical specialists in Gauteng (42.2%) and the Western Cape (27.4%) in 2016. At the time, these provinces accommodated only, respectively, about two in every 10 and one in every 10 of the country’s public sector-dependent people.^17^

The National HRH Plan of the NDoH (2006)^45^ recommended an increase in the undergraduate medicine programme output from 1300 to 2400 graduates per annum. This growth never occurred as anticipated. South Africa thus entered into a bilateral agreement with Cuba to alleviate acute MP shortages in the public sector. About 3300 medical students were sent to this country for training of whom 657 have graduated since 2004.^8,46^ Econex’s study on Doctor Shortages study (2015)^26^ opined that even the South African private sector may have too few MPs, that is, there were just 2.5 public and 9.2 private sector MPs per 1000 population in 2013 – the combined average was 6.0 per 1000 population, compared to 15.2 per 1000 population globally.

The HRH Strategy (2011)^9^ was uncertain how many MPs emigrate, but between 2004 and 2009, 17% of MPs who qualified did not report for community service. In 2016, the media^47^ reported that between 70% and 80% of MPs preferred not to work for the state. The same media stated that although foreign MPs were eager to work in the country, bureaucratic challenges often prevented this. As articulated in the Policy on Recruitment and Employment of Foreign Health Professionals (ca. 2006),^22^ the government maintains a general principle that no foreign HCW may seek employment without a formal letter of invitation or employability. There is also need for better integration of foreign MPs into the public sector: ‘the level of fragmentation characterising the milieu, with tensions along language, ethnic, racial, gender, hierarchical and professional lines, was adding to foreigners’ difficult integration’.^48^

### Clinical specialists

The HRH Strategy (2011)^9^ indicated that a shortage of 5677 medical specialists would occur in 2015, but nevertheless expected that the DCST in each district will consist of four CSs (paediatrician, family physician, obstetrician and gynaecologist, and anaesthetist), as well as an advanced midwife, advanced paediatric nurse and advanced PHC nurse. The cross-disciplinary approach has proved to be successful if the DCST team shares a common vision and has a work plan to guide the priorities and facility support visits.^49^ The purpose of DCSTs is to enhance clinical governance at the lowest level of the health system to improve the quality of maternal, neonatal, child and women’s health (MNCWH) care in South Africa, through strengthening four pillars of clinical governance: (1) clinical effectiveness (use of evidence-based protocols); (2) clinical risk management (safe care without harm); (3) professional development and management and (4) creating demand and improving accountability for MNCWH.^50^

Oboirien et al.’s^15^ findings revealed that although the DCST was considered to be an important innovation, with high expectations about their role in PHC strengthening, the extent of the DCSTs’ integration was highly dependent on existing capacity, flexibility and ‘matching of expected roles to resources’. Also, role indistinctness and conflict at the system-wide level was experienced in the implementation of the DCST strategy, suggesting a need for more effective management of implementation systems.^51^ In 2014, Voce et al. reported that only a small proportion of districts had a fully constituted DCTS, but that teams, dyads (where present) and individuals were fulfilling key responsibilities towards improving the quality of clinical services.^52^ As of March 2015, besides various measures having been put into place to measure effective implementation of the DCSTs, 49 out of the 52 districts in South Africa had appointed at least a minimal team (a dyad – nurse/doctor pair in one discipline).^53^

In 2014, a Ministerial Task Team (MTT)^54^ recommended that a conditional grant should be established to cover maternal services that include a line item covering DCSTs to ensure their sustained public sector funding. The MTT also recommended that HRH policies should be revisited to improve health professional retention and CS coverage in rural areas, including requirements for community service for specialist registration, rotation as part of registrar training and periods of deployment in regional hospitals in underserved areas. However, as far as could be determined, data on DCST and CS numbers and distribution are not available, thus ruling out assessment of the progress made in implementing this key HRH intervention to address high maternal mortality in South Africa.

In 2018, Feucht et al. reported that in confined areas in South Africa, DCSTs were generating innovations focused on key service delivery improvements, with notable outputs and with documented reductions in institutional deaths and fatality rates over time.^50^ Along with concurrent implementation of a range of evidence-based interventions to achieve these reductions, the improved processes and skills that the DCST innovations introduced were ‘obvious enabling success factors’. However, observed weaknesses in reviewed DCST initiatives included that quality standards such as respect and dignity, communication and emotional support were not addressed. These authors recommended that the development and scale-up of DCST innovations needed to be institutionalised and have to include effective support and action from the relevant health managers.

## Summary

Key observations from this review are the following: (1) Despite policy recognition that CHWs have a critical role in comprehensive PHC, there are few data available on their supply and distribution in South Africa. Available data suggest wide disparity in the provincial supply of CHWs. (2) Professional nurse training is currently severely compromised by non-alignment of policies and statutory processes. (3) Because the role and regulation of MLWs have not been finalised, their development and wider deployment are being delayed. This means that an important HRH strengthening opportunity is being missed. (4) The allocation of MPs in the public sector is misaligned and is not adequately addressing PHC challenges. This is because there is no consensus and national policy on how and where best to place doctors to best meet the demand for medical services among those most in need. (5) For CSs to make an impact in the public sector, there should be at least one full complement of DCSTs in every district. At the moment there is a real threat that PHC re-engineering will not be achieved if the rural–urban and public–private divide among CSs distribution is not closed.

Gaps and areas for research in terms of HRH requirements for successful NHI implementation and PHC re-engineering include (1) further clarification of multi-disciplinary team compositions, roles and responsibilities; (2) social factors affecting health professionals in rural areas and health professional retention for the DHS; (3) clinical governance roles of MPs in their interaction with other HCWs and communities; (4) resource and support needs of WBOTs and CHWs and (5) progress made in establishing the DCSTs.

Novel models to calculate staffing norms for new policies and services are constantly being developed. This review concentrated on the most recent available information; however, techniques to calculate optimal staff categories, combinations and activities differ and are variably used in different settings. A further limitation of this review is that it is not based on primary research. Future research could consider wider consultation with key informant groups across the public and private sectors to report their experience and perspectives.

## Conclusion

The NDP (2012), PHC re-engineering (2011), HRH (2011) and NHI (2017) policies are well drafted with beneficent intent. These policies provide the overall vision and strategies for South Africa to pursue equitable health care for all. Nonetheless, the latest HRH policies fall short in that there is disjuncture between them and that they are not translated into synchronised strategic plans to produce sufficient numbers and more equitable distribution of HCWs. Aligning the policies and expediting their strategic implementation are essential to reach the desired goal of UHC through NHI and PHC re-engineering, and to fulfil the 2030 NDP vision of reduced health inequality and inequity.
