# Corrigendum: Towards national health insurance: Alignment of strategic human resources in South Africa

**DOI:** 10.4102/phcfm.v13i1.3187

**Published:** 2021-10-20

**Authors:** Nathaniel Mofolo, Christo Heunis, Gladys N. Kigozi

**Affiliations:** 1 School of Clinical Medicine, University of the Free State, Bloemfontein, South Africa; 2 Centre for Health Systems Research and Development, University of the Free State, Bloemfontein, South Africa

In the version of this article initially published, Mofolo N, Heunis C, Kigozi GN. Towards national health insurance: Alignment of strategic human resources in South Africa. Afr J Prm Health Care Fam Med. 2019;11(1), a1928. https://doi.org/10.4102/phcfm.v11i1.1928, on page 4, the last paragraph under the heading ‘Mid-level workers’ was given incorrectly. The correct paragraph should read:

‘Despite the appreciation for CAs,^28,41,42,43^ this new cadre has also generated serious concerns. According to a 2017 Parliamentary Monitoring Group report,^44^ CAs were “ignored in rural areas” and “poached by the United Kingdom”. In 2016, the scope of practice for a CA was promulgated by the Health Professions Council of South Africa, and signed off by the Health Minister. However, the CA is yet to be listed as authorised prescriber which impedes their efficient functioning. With appropriate support, supervision and recognition CAs will make a significant contribution to the success of the NHI, especially if more HEIs train CAs.’,

instead of:

‘Despite the appreciation for CAs,^28,41,42,43^ this new cadre has also generated serious concerns. According to a 2017 Parliamentary Monitoring Group report,^44^ CAs were “ignored in rural areas” and “poached by the United Kingdom”. In 2016, the scope of practice for a CA was promulgated by the Health Professions Council of South Africa, but has yet to be signed off by the Health Minister. This implies that it remains difficult to define MLWs’ roles within multi-disciplinary teams.’

On page 4, the first paragraph under the heading ‘Medical practitioners’ was given incorrectly. The correct paragraph should read:

‘Although the NDP (2012)^7^ envisaged “[*i*]ncreasing the numbers of medical professionals”, the HRH Strategy (2011)^9^ indicated that a shortage of 3930 MPs would occur in 2015. The WHO’s Global Health Observatory (2018)^20^ shows that over 45% of member states, including South Africa, have less than 1.0 MPs per 1000 population. Compared to the other BRICS countries, the supply of MPs at 0.82 per 1000 population in South Africa in 2016 was somewhat higher than India at 0.76 per 1000 population (2016), but substantially lower than the 4.01 per 1000 population in the Russian Federation (2016), 1.86 per 1000 population in Brazil (2013) and 1.78 per 1000 population in China (2015). Thereupon, as measured by healthy life expectancy at birth for both sexes in 2015, the need for MPs was highest in South Africa (62.9 years) and lowest in the Russian Federation (70.5 years) and Brazil (75.0 years).’,

instead of:

‘Although the NDP (2012)^7^ envisaged “[i]ncreasing the numbers of medical professionals”, the HRH Strategy (2011)^9^ indicated that a shortage of 3930 MPs would occur in 2015. The WHO’s Global Health Observatory (2018)^20^ shows that over 45% of member states, including South Africa, have less than 10 MPs per 1000 population. Compared to the other BRICS countries, the supply of MPs at 8.2 per 1000 populationin South Africa in 2016 was somewhat higher than India at 7.6 per 1000 population (2016), but substantially lower than the 39.8 per 1000 population in the Russian Federation (2015), 18.5 per 1000 population in Brazil (2013) and 18.1 per 1000 population in China (2015). Thereupon, as measured by healthy life expectancy at birth for both sexes in 2015, the need for MPs was highest in South Africa (62.9 years) and lowest in the Russian Federation (70.5 years) and Brazil (75.0 years).’

On page 5, the second last paragraph under the heading ‘Medical practitioners’ was given incorrectly. The correct paragraph should read:

‘The National HRH Plan of the NDoH (2006)^45^ recommended an increase in the undergraduate medicine programme output from 1300 to 2400 graduates per annum. This growth never occurred as anticipated. South Africa thus entered into bilateral agreement with Cuba to alleviate acute MP shortages in the public sector. About 3300 medical students were sent to this country for training of whom 657 have graduated since 2004.^8,46^ Econex’s study on Doctor Shortages study (2015)^26^ opined that even the South African private sector may have too few MPs, that is, there were just 0.25 public and 0.93 private sector MPs per 1000 population in 2013 – the combined average was 0.60 per 1000 population, compared to 1.52 per 1000 population globally.’,

instead of:

‘The National HRH Plan of the NDoH (2006)^45^ recommended an increase in the undergraduate medicine programme output from 1300 to 2400 graduates per annum. This growth never occurred as anticipated. South Africa thus entered into bilateral agreement with Cuba to alleviate acute MP shortages in the public sector. About 3300 medical students were sent to this country for training of whom 657 have graduated since 2004.^8,46^ Econex’s study on Doctor Shortages study (2015)^26^ opined that even the South African private sector may have too few MPs, that is, there were just 2.5 public and 9.2 private sector MPs per 1000 population in 2013 – the combined average was 6.0 per 1000 population, compared to 15.2 per 1000 population globally.’

This correction does not alter the study’s findings of significance or overall interpretation of the study’s results. The authors apologise for any inconvenience caused.

